# Women in vascular surgery: a brief analysis of the Brazilian profile

**DOI:** 10.1590/1677-5449.011317

**Published:** 2018

**Authors:** Fernanda Costa Sampaio Silva, Monique Magnavita Borba da Fonseca Cerqueira, Bárbara Beatriz Couto Ruivo, Marita von Rautenfeld

**Affiliations:** 1 Centro Médico Hospital da Bahia – HBA, Departamento de Angiologia e Cirurgia Vascular, Salvador, BA, Brasil.; 2 Secretaria de Saúde do Estado da Bahia – SESAB, Departamento de Angiologia e Cirurgia Vascular, Salvador, BA, Brasil.; 3 Santa Casa de Misericórdia da Bahia, Departamento de Angiologia e Cirurgia Vascular, Salvador, BA, Brasil.; 4 Dr. Consulta, Departamento de Cirurgia Vascular e Flebologia, São Paulo, SP, Brasil.; 5 Hospital São Camilo-Santana – HSC, Departamento de Angiologia e Cirurgia Vascular, São Paulo, SP, Brasil.; 6 Hospital Dia Rede Hora Certa-Penha, Departamento de Angiologia e Cirurgia Vascular, São Paulo, SP, Brasil.; 7 Samar Serviços Médicos – SAMAR, São Paulo, SP, Brasil.

**Keywords:** women, vascular surgery, professional women, female physicians

## Abstract

**Background:**

There has been a striking increase in female participation in the Brazilian and worldwide medical contingent, but the number of female surgeons does not follow the same trend. In addition to quantitative analysis, there is a need for further study of the determinants of choice of surgical specialty and the characteristics of professional practice.

**Objectives:**

To outline the profile of female vascular surgeons in Brazil in terms of demographic characteristics, qualifications, scientific engagement, and labor market integration.

**Methods:**

A survey was made available online for 30 days and its link was widely circulated among female vascular surgeons. At the end of data collection, 101 valid questionnaires had been returned and their data were tabulated in Microsoft Excel spreadsheets for simple descriptive analysis.

**Results:**

The profile traced was of women aged up to 45 years who have been working in the area for up to 10 years. They were predominantly trained in medical residencies or internships recognized by the specialty board. Venous surgery, Esthetic Phlebology and Vascular Ultrasound were the major fields of activity for female vascular surgeons. Although they hold degrees and author scientific publications, the proportion of leadership positions held by women remains low. More than 64% of the participants reported feeling undervalued because they were women.

**Conclusions:**

Despite the limitations of the study population, this preliminary study confirms the idea that female vascular surgeons demonstrate continuous dedication to practicing their specialty and sets a precedent so that further studies can investigate the professional practice of female vascular surgeons in greater detail, stimulating discussion of gender inequalities in medical practice.

## INTRODUCTION

 For a long period in history, medicine was a prohibited profession for women. The first female physician to receive her diploma from a Brazilian university was Rita Lobato Lopes, in 1887. 1 Since then, there has been considerable growth in female participation in the Brazilian and global medical contingent. Recent data from the Brazilian Medical Demograph 2 showed that 54% of physicians in São Paulo under the age of 35 are female. 

 However, although the numbers of women in medicine are growing progressively, the surgical specialties remain a predominantly male niche all over the world. In 1994 and 1995, only 13% of physicians in the surgical specialties in France were female, in contrast with 21% in pediatrics. 3 For a very long time, surgery has been an essentially male specialty, because of the way physicians are recruited and because of technical and cultural characteristics. 

 Franco and Santos conducted a retrospective survey in 2010, 4 using data from the Brazilian College of Surgeons for 1950 to 2000, showing that there was a significant increase in the number of female surgeons in the 1980s and 1990s; but the female gender’s share in the surgical specialties has still not kept pace with the general tendency of growth. 5 The authors identified factors that contribute to the small number of women in surgery, including difficulties to be overcome, a certain lack of self-confidence, and scarcity of models of successful women who can be used as references, in addition to problems caused by the absence of institutional support for physicians who are mothers. 

 In the field of vascular surgery, data published in 2017 by Sadiq et al. 6 show that only 14.6% of professionals currently practicing in the United States are women. Furthermore, proportionally, female vascular surgeons occupy fewer leadership roles and have fewer academic publications. 

 Similar disparities are observed in Brazil, where 87% of vascular surgeons were men in 2004 7 and, in 2015, 78.8% of vascular surgeons in the state of São Paulo were men. 2 Preliminary results of the vascular census that is being conducted by the Brazilian Society of Angiology and Vascular Surgery (SBACV) indicate that there are 3,209 vascular surgeons in the country. However, this survey has not analyzed sex distribution. 8

 In addition to quantitative analysis, some research has been conducted with the intention of assessing qualitative aspects of surgical practice, attempting to establish the determinant factors in choice of specialty by professionals of both sexes. 9 The present study was conducted with the objective of tracing the distribution of female vascular surgeons in Brazil and their practice profiles, stimulating discussion to promote female integration in the profession. 

## METHODS

 This is a preliminary, cross-sectional, descriptive study conducted with the intention of delineating the profile of female Brazilian vascular surgeons. The methodology employed conforms to the principles set out in Guidelines on Good Publication Practice, produced by the Committee on Publication Ethics (COPE). 10 An electronic questionnaire with 15 questions was developed by the authors and made available on-line for 30 days via the survio.com portal. The link to the survey was divulged using telephone lists, medical groups on the WhatsApp messaging app, and social networks, to practicing and future (still in training) female vascular surgeons, irrespective of membership of the SBACV, in order to reach a wide-ranging sample. In total, 293 female vascular surgeons were contacted via the WhatsApp groups “Vasculadies” and “ClubVas”, which are online fora for socialization and discussion of clinical cases and scientific articles among women in the specialty. The database of the CANU system (813 women), in the manner that it is made available on the Brazilian Society of Angiology and Vascular Surgery website, only offers the ability to search by name or postcode and does not provide a list of specialists with their respective electronic addresses. This database could not therefore be used for distribution of the questionnaires. Question 1 on the instrument requested the participant to provide their name and Regional Medical Council registration number (CRM) and questionnaires for which the name provided did not correspond to the CRM, duplicate questionnaires, and questionnaires with false names were excluded. As such, Question 1 is not used in analysis of the results and no data that could identify the informants are used. The questionnaire was developed based on similar studies by Umoetok et al. 5 and Kwong et al., 9 adapted for the Brazilian setting. The order of the questions was designed to enable a coherent discussion of the results and they were distributed as follows: Question 1 collects participants’ medical board registration details; questions 2 to 4 collect demographic data; questions 5 to 7 cover length of experience and elements of professional training and qualifications; questions 8 to 11 cover data on position in the employment market; questions 12 to 14 assess management positions and degree of involvement in academic and scientific matters; and, finally, Question 15 measures each participant’s personal satisfaction as a woman practicing the specialty. After collection, data were tabulated in a Microsoft Excel spreadsheet for simple descriptive analysis. The questionnaire is shown in Chart 1 . It should be reiterated that this is a preliminary study, conducted to encourage debate on a current subject that is discussed little in the vascular community. It is estimated that the SBACV currently has around 800 women registered on its national specialty register, so the sample size needed to permit statistical inferences would be around 260 questionnaires from society members, considering a maximum margin of error of 4% and a 95% confidence level. A larger study is in development, for which the institutional support of the SBACV will be essential to involving a more representative number of participants. 

**Chart 1:** Questionnaire “Women in vascular surgery: a brief analysis of the Brazilian profile”.

Question 1. Provide your name and CRM registration number
Question 2. Nationality: ___Brazilian __other (please specify _______)
Question 3. Which state do you practice in? __Amazonas __Ceará __Pernambuco __ Alagoas __Paraíba __Sergipe __ Bahia __Tocantins __Goiás __Mato Grosso __Mato Grosso do Sul __Acre __Espírito Santo __Minas Gerais __Rio de Janeiro __São Paulo __Paraná __Santa Catarina __Rio Grande do Sul __Distrito Federal __Amapá __Roraima __Rondônia __Pará __Rio Grande do Norte __Maranhão __Piauí
Question 4. How old are you? __25-35 years __36-45 years __46-55 years __56-65 years __more than 65 years old
Question 5. Time practicing the specialty: __less than 5 years __6 to 10 years __11 to 20 years __more than 20 years
Question 6. Have you taken a specialization course? __No, but I worked as a vascular physician and took a board certification exam afterwards __Yes, medical residency __Yes, an internship recognized by the SBACV __I am still training __None of the above
Question 7. Do you hold any of the following specialist qualifications? __Specialist in angiology __Specialist in vascular surgery __Certification in vascular ultrasound with Doppler __Certification in radio angiology or endovascular surgery __Two or more of the qualifications above __No specialist qualification
Question 8. You practice... __Exclusively in the public healthcare system __Exclusively in private medicine __Both
Question 9. You work more hours __Preferably in a consulting room/clinic __Preferably in a hospital
Question 10. In what type of consulting room/clinic do you work? __Own __Sublet __In partnership with owners / on a percentage basis __Other (please specify)
Question 11. Currently, what is your principal field of activity? __Arterial surgery __Venous surgery __Endovascular surgery __Vascular ultrasound __Esthetic phlebology __A mixture of activities, unable to specify
Question 12. Have you ever held one of the following management roles? __Yes, within the SBACV __Yes, as supervising physician of a service __No, I have never held a management position __Yes, a different role (please specify)
Question 13. Have you ever taken part in any of the following academic activities? __Presentation at symposium/congress __Round table speaker at symposium/congress __Both __Neither of those described above
Question 14. Have you ever had an academic paper published? __Yes __No
Question 15. At any point during your career have you ever felt undervalued or at a disadvantage because you are a woman? ___Yes __No

## RESULTS

**Figure 1:**
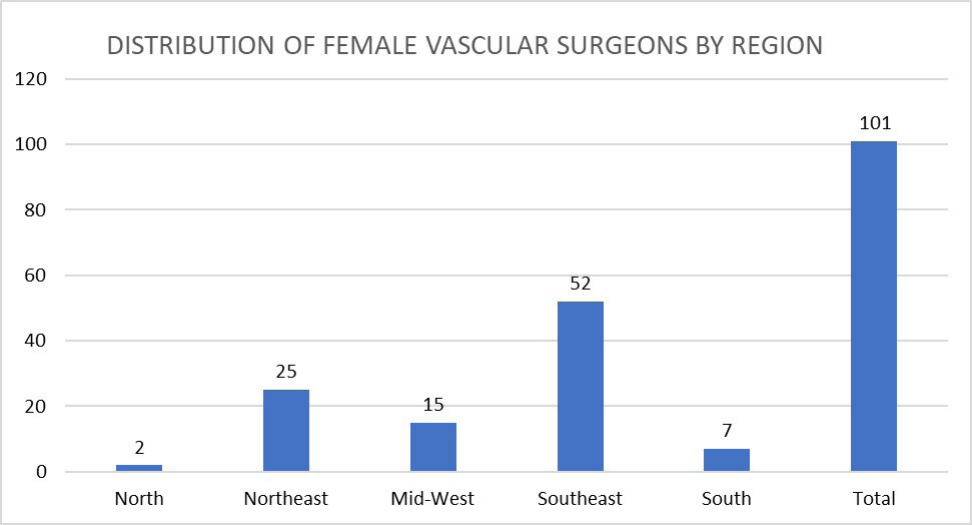
Graph illustrating distribution of female vascular surgeons in Brazil by region. Values are in absolute numbers.

 At the end of 30 days of data collection, 106 of the 293 questionnaires sent out had been completed, five of which were excluded because the data provided in answer to Question 1 were invalid. Just one of the 101 valid questionnaires reported a nationality other than Brazilian (Colombian). The category with the greatest number of responses to the third question (“Which state do you practice in?”) was São Paulo (n = 43), followed by Bahia (n = 11). Figure 1 illustrates the distribution of participants across Brazil’s five administrative regions. 

 Question 4 classified female vascular surgeons by age group, revealing that 46.5% were 25-35 years of age, 43.6% were 36-45 years old, and less than 10% were over the age of 46. Question 5, “Time practicing the specialty” revealed that 35.6% of the participants had been practicing in the field for less than 5 years, 36.6% for 6 to 10 years, 23.8% for 11 to 20 years, and 4% had been in the profession for more than 20 years. 

**Figure 2:**
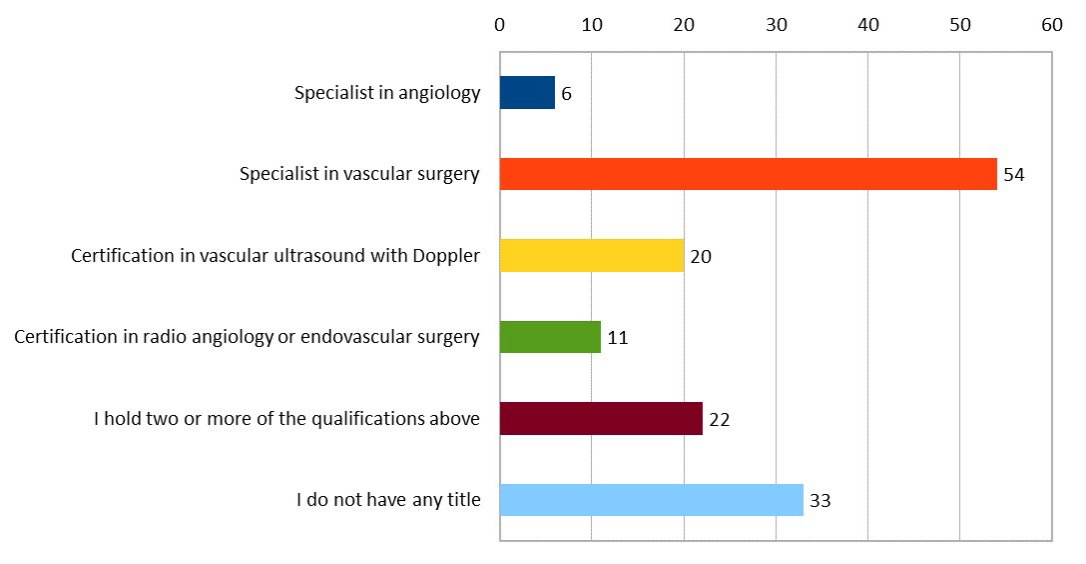
Graph illustrating participants’ specialist qualifications. Values are in absolute numbers. Note that the question allowed more than one response per participant to be selected.

 In response to Question 6 (“Have you taken a specialization course?”), 76.2% stated they had completed a medical residency, 18.8% an internship recognized by the SBACV, and 4% were still in training, while 1% responded “None of the above”. The results for question number 7 (“Do you hold any of the following specialist qualifications?”) are illustrated in Figure 2 . 

 With relation to the economic aspects of professional practice, in response to item 8, three participants stated that they worked exclusively in the public healthcare system, 29 worked exclusively in private medicine, and 69 worked in both systems. In response to Question 9, 63.4% of the participants indicated that they preferably worked more hours in a consulting room/clinic. Item 10 asked in what type of institution participants conducted their clinical practice, to which 39 participants stated they had their own practice, 24 worked in a sublet clinic, and 41 in a partnership/percentage agreement with the owners of a consulting room/clinic, while 11 replied “other”, specifying in their own words that they worked in a clinic owned by an insurer (n = 2), in a national health service (SUS) clinic (n = 2), or as part of a medical residency (n = 2), or stated that they do not have a consulting room (n = 2). 

**Figure 3:**
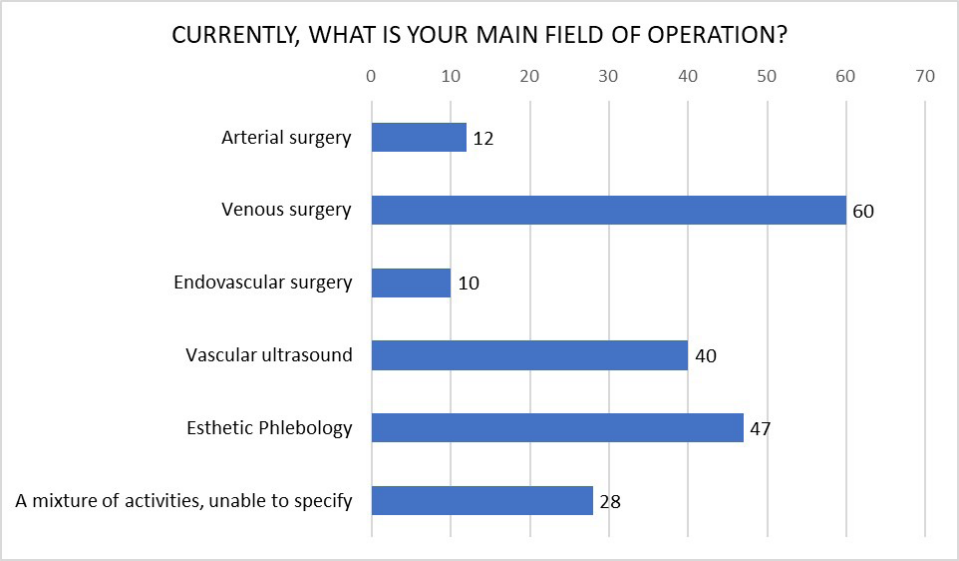
Graph illustrating diversity of female participation in the major branches of the specialty. Values are in absolute numbers. Note that the question allowed more than one response per participant to be selected.

Figure 3 illustrates the results for question 11 (“Currently, what is your principal field of activity?”). 

**Figure 4:**
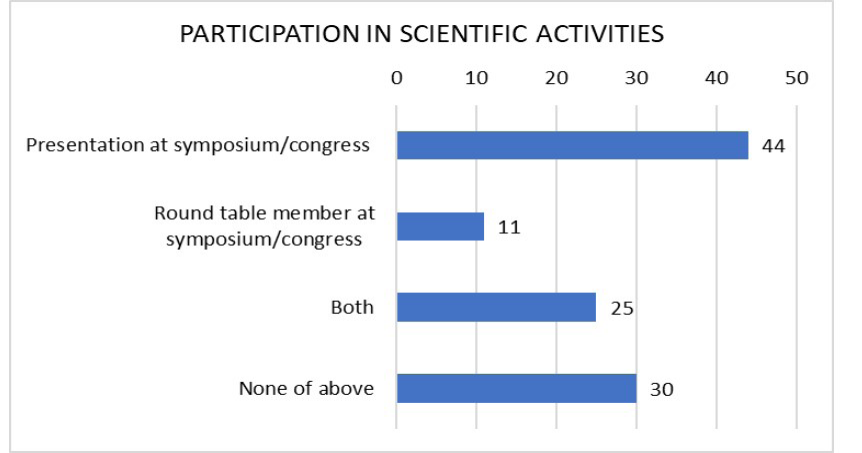
Level of participants’ scientific involvement. Values are in absolute numbers. Note that the question allowed more than one response per participant to be selected.

 To item 12, “Have you ever held one of the following management roles?”, 7.9% responded “yes, within the SBACV”; 30.7% had been supervising physicians at a service; 5.9% had held other roles, described in their own words as technical director of healthcare institutions (n=3); and 62.4% had never held a management position. Figure 4 illustrates the data from Question 13 (“Have you ever taken part in any of the following scientific activities?”). In answer to question 14, 64.4% of the participants stated they had published a scientific paper. 

 Finally, 64.4% of those interviewed replied “Yes” to Question 15, “At any point during your career, have you ever felt undervalued or at a disadvantage because you are a woman?” 

## DISCUSSION

 The data collected show that, in the sample studied here, the profile of female vascular surgeons in Brazil is of women up to 45 years of age who have been practicing in the field for a maximum of 10 years. The young age of those interviewed corresponds to the relatively recent interest of women in surgical specialties, as shown by Troppmann et al. 11 History is marked by exclusion of women, and not only in medical settings. Anthropologically, men had always been considered more capable of using tools and instruments of production, giving them domination over women. 4 In order to embark on a surgical career, it is necessary to be able to deal with situations of great severity, which fits a global tendency with respect to training of a new generation of surgeons: young people avid for challenging situations and a working environment in which they can express their convictions. 12 , 13 Socially, males are predisposed to and are accepted performing activities of leadership and combat and also activities that demand greater physical strength, which can be the case in the field of surgery. 14 , 15 All of these characteristics have been socially constructed over many years, resulting in an ideological position that male physicians would be more suited to roles in the field of surgery; 16 but this professional profile is being observed ever more frequently among women. 

 In the present study, there was a predominance of participants from the Southeast region of Brazil, especially the state of São Paulo, which partially reflects the concentration of medical schools in this region. The proportion of participants is smaller where there are also lower concentrations of specialist physicians. Women who qualify help to build up the local workforce, contributing to a better distribution of physicians throughout Brazil. 17

 Although the majority of the female surgeons have completed a medical residency or internship recognized by the SBACV and more than half have qualifications from specialist medical boards, almost 2/3 have never held a management position, and those who have had this opportunity have done so within training services. Over the 66-year history of the SBACV, which was previously known as the Brazilian Angiology Society (SBA), it has only had two female presidents: Dr. Merisa Garrido, in 1990 and 1991, and Dr. Maria Elisabeth Rennó de Castro Santos, in 2000 and 2001. 18 Furthermore, during the last six two-year terms, only six of the Society’s 84 positions, including directors and vice-directors, have been held by women and during the last three terms not a single woman has been involved in managing the Society. The low number of women on the Society’s board confirms the perception that women are under-represented in leadership roles, providing evidence that, despite the increase in the number of female surgeons, their progress in advancing into the better-paid and higher-prestige roles is still limited. 5 , 19 Disadvantages such as these, which are borne throughout the career path, were felt by 64% of those interviewed. 

 In contrast, almost 2/3 of the professionals who completed the questionnaire stated that they had had a scientific paper published and more than half stated that they had participated in a congress as a speaker or member of a round table. Indeed, there is engagement in academic activities, which could be developed further if there was an opportunity to take part in research, publications, and presentations during training in vascular surgery. 20

 The majority of the female surgeons combine work in the public and private healthcare systems, spending a large proportion of their working hours in clinical consultations. A little more than a third of the female vascular surgeons have their own practices, and venous surgery, esthetic phlebology, and vascular ultrasound are their main occupations. Just 12 participants stated that arterial surgery was their principal field of activity and just 10 dedicated themselves to endovascular surgery as a priority. It is known that the factors related to the small number of women in arterial and endovascular surgery include difficulties with reconciling their profession with their personal lives, including marriage and maternity. 21 Added to this is the understanding that choice of field of activity within the specialty is not always a question of personal choice. It cannot be ignored that the preference for enrolling male professionals in arterial surgery teams constitutes a cultural barrier that is yet to be overcome. As described by Franco and Santos, 4 personal characteristics seen as necessary to work in surgery, such as leadership, self control, willingness to question, a strong personality, and a certain degree of aggression are seen as inherent to the male but strange in the female personality. 

 On the other hand, taking into account incorporation of new technologies in the field of phlebology, and the preference for venous and esthetic surgery, beyond offering an escape route because this field offers the possibility of combining quality of life and flexibility of working hours, this may also offer a promising strategy for integration of women in the employment market. Since expansion of the field of phlebology demands continuous updating and ongoing education, it drives the emergence of new leading scientific positions, which can perfectly well be occupied by women. Thus, with good prospects for academic and financial growth, there is a potential incentive for promotion of successful careers, reversing the paradigm illustrated by the last question posed in this study. 

 Globally, understanding of the existence of factors that limit full entry of woman into surgical careers has led to the emergence of certain movements to encourage integration and mutual cooperation between female surgeons, such as Women in Thoracic Surgery (WTS) 22 and the Association of Women Surgeons (AWS). 23 In Brazil, the Brazilian Society of Cardiovascular Surgery (SBCCV) has been conducting motivational projects such as the video *Ballet de Mãos – As Cirurgiãs Cardiovasculares* [A Ballet of Hands – The Female Cardiovascular Surgeons], 24 valuing and praising the presence of women in the specialty. While still incipient, proposals such as this can become a driving force for changes to the scenario of woman in surgical specialties. 

## LIMITATIONS

 The number of participants interviewed in this study is small (n = 101), in relation to the population of female vascular surgeons in Brazil. Therefore, the results reported here cannot be generalized and are applicable only to the sample selected. Additionally, there was selection bias, since questionnaires were not sent to the entire population of female vascular surgeons in Brazil, but to women who were part of the authors’ network of contacts. 

## CONCLUSIONS

 The future of vascular surgery depends on training well-qualified professionals, of both sexes. However, considering the impact of female participation in the workforce is of fundamental importance for development of strategies to ensure good performance in the specialty in future generations. This study confirms the idea that female vascular surgeons demonstrate continuous dedication to practicing their specialty and strive for academic growth. Although this article presents a preliminary study, we believe that it sets a precedent so that further studies can investigate the professional practice of female vascular surgeons in greater detail, with larger samples that enable statistical inferences, fostering discussion of their characteristics and of gender inequalities within the specialty, in line with the global tendency in many other fields of medical knowledge. 
