# Supplementary material for: Women in vascular surgery: a brief analysis of the Brazilian profile
Source: J Vasc Bras. 2018 Apr-Jun;17(2):128–35. doi: 10.1590/1677-5449.011317 (PMC6205712; doi:10.1590/1677-5449.011317)
Supplement: Translation

# Translation: Mulheres na cirurgia vascular: uma breve análise do perfil brasileiro

**Authors:** Fernanda Costa Sampaio Silva, Monique Magnavita Borba da Fonseca Cerqueira, Bárbara Beatriz Couto Ruivo, Marita von Rautenfeld

**Affiliations:** 1 Centro Médico Hospital da Bahia – HBA, Departamento de Angiologia e Cirurgia Vascular, Salvador, BA, Brasil.; 2 Secretaria de Saúde do Estado da Bahia – SESAB, Departamento de Angiologia e Cirurgia Vascular, Salvador, BA, Brasil.; 3 Santa Casa de Misericórdia da Bahia, Departamento de Angiologia e Cirurgia Vascular, Salvador, BA, Brasil.; 4 Dr. Consulta, Departamento de Cirurgia Vascular e Flebologia, São Paulo, SP, Brasil.; 5 Hospital São Camilo-Santana – HSC, Departamento de Angiologia e Cirurgia Vascular, São Paulo, SP, Brasil.; 6 Hospital Dia Rede Hora Certa-Penha, Departamento de Angiologia e Cirurgia Vascular, São Paulo, SP, Brasil.; 7 Samar Serviços Médicos – SAMAR, São Paulo, SP, Brasil.

**Keywords:** mulheres, cirurgia vascular, mulheres profissionais, médicas

## Abstract

**Contexto:**

O aumento da participação feminina no contingente médico brasileiro e mundial tem sido notório; entretanto, o número de mulheres cirurgiãs não acompanha a mesma tendência. Além da análise quantitativa, os fatores determinantes da escolha pela especialidade cirúrgica e as características da atuação profissional necessitam de mais estudos.

**Objetivos:**

Delinear o perfil das cirurgiãs vasculares brasileiras no que diz respeito às características demográficas, grau de qualificação, engajamento científico e integração ao mercado de trabalho.

**Métodos:**

Um questionário eletrônico foi disponibilizado on-line durante 30 dias e teve seu link amplamente divulgado entre cirurgiãs vasculares. Após coleta, retornaram 101 questionários válidos, cujos dados foram tabulados em planilhas do Microsoft Excel para análise descritiva simples.

**Resultados:**

O perfil encontrado foi de mulheres com até 45 anos de idade que atuam na área por no máximo 10 anos. A formação foi predominantemente por meio de residência médica ou estágio reconhecido pela sociedade da especialidade. Cirurgia venosa, flebologia estética e ecografia vascular mostraram-se como principais campos de atuação para cirurgiãs vasculares. Apesar de possuírem titulação e publicações científicas, a sua representatividade nos cargos de liderança permanece baixa. Mais de 64% das participantes referiram sentimento de desvalorização profissional devido ao fato de serem mulheres.

**Conclusões:**

Apesar das limitações da população estudada, este estudo preliminar corrobora com a ideia de que as cirurgiãs vasculares demonstram uma contínua dedicação à especialidade e abre um precedente para que novas pesquisas acessem os pormenores dessa atuação profissional, fomentando a discussão sobre as desigualdades entre os gêneros no exercício da medicina.

## INTRODUÇÃO

 Por um longo período na história, a medicina foi profissão proibida para as mulheres. A primeira médica diplomada em universidade brasileira foi Rita Lobato Lopes, em 1887 1 . Desde então, tem sido notório o crescimento da participação feminina no contingente médico nacional e mundial. Dados da Demografia Médica Brasileira 2 apontam que, no estado de São Paulo, 54% dos médicos na faixa etária até 35 anos são do sexo feminino. 

 Embora a medicina conte com um número progressivamente maior de mulheres, as especialidades cirúrgicas permanecem como um nicho predominantemente masculino em todo o mundo. Entre os anos de 1994 e 1995, as especialidades cirúrgicas contavam com apenas 13% de médicas na França, contrastando com a presença de 21% de mulheres na área de pediatria 3 . A cirurgia é, de longa data, uma especialidade essencialmente masculina, devido ao tipo de recrutamento dos médicos, às características técnicas e também às características culturais. 

 O levantamento realizado por Franco e Santos 4 em 2010, utilizando dados do Colégio Brasileiro de Cirurgiões entre 1950 e 2000, registrou um aumento significativo no número de cirurgiãs nas décadas de 1980 e 1990; entretanto, a representatividade do gênero feminino dentro das especialidades cirúrgicas não acompanha a mesma tendência geral de crescimento 5 . Para as autoras, dentre os fatores que contribuem para o pequeno número de mulheres no campo da cirurgia estão a existência de dificuldades a serem vencidas e uma certa falta de autoconfiança e de modelos de sucesso feminino que possam ser utilizados como referência, além dos problemas advindos da falta de suporte institucional às mães médicas. 

 No campo da cirurgia vascular, conforme dados levantados por Sadiq et al. 6 em 2017, atualmente, nos Estados Unidos, as mulheres correspondem a apenas 14,6% dos profissionais atuantes. Além disto, proporcionalmente, as cirurgiãs vasculares ocupam menos espaço nos cargos de liderança e na produção acadêmica do país. 

 Disparidades semelhantes são observadas no Brasil, onde os homens representavam 87% dos cirurgiões vasculares no território nacional até o ano de 2004 7 e 78,8% no estado de São Paulo em 2015 2 . Resultados preliminares do censo vascular, que está sendo elaborado pela Sociedade Brasileira de Angiologia e Cirurgia Vascular (SBACV), apontam a existência de 3.209 cirurgiões vasculares no país. Entretanto, nesse levantamento, a distribuição entre gêneros não foi aprofundada 8 . 

 Além da análise quantitativa, algumas pesquisas têm sido conduzidas com o propósito de avaliar os aspectos qualitativos da prática cirúrgica, procurando estabelecer os fatores determinantes na escolha da especialidade por profissionais de ambos os sexos 9 . Este estudo foi realizado com o intuito de compreender a distribuição das cirurgiãs vasculares no Brasil, bem como seu perfil de atuação, fomentando discussões que favoreçam a integração feminina na área. 

## MÉTODOS

 O presente estudo preliminar, transversal e descritivo foi realizado no intuito de delinear o perfil da cirurgiã vascular brasileira. A metodologia aplicada seguiu os preceitos contidos no documento *Guidelines on good publication practice*, elaborado pelo Committee on Publication Ethics (COPE) 10 . Um questionário eletrônico com 15 perguntas foi elaborado pelas autoras e disponibilizado on-line através do portal survio.com, durante um período de 30 dias. O link da pesquisa foi divulgado através de contatos telefônicos, grupos médicos do aplicativo de mensagens WhatsApp e redes sociais de cirurgiãs vasculares em exercício ou em formação, associadas ou não à SBACV, de forma a abranger uma amostragem ampla. Foram contatadas 293 cirurgiãs vasculares que participam dos grupos “Vasculadies” e “ClubVas” no WhatsApp, os quais se propõem a serem espaços online de socialização, discussão de casos clínicos e artigos científicos entre as mulheres da especialidade. O banco de dados do sistema CANU (813 mulheres), na forma como disposto no site da Sociedade Brasileira de Angiologia e Cirurgia Vascular, permite apenas a busca pelo nome do profissional ou pelo Código de Endereçamento Postal (CEP), não disponibilizando uma lista de especialistas com seus respectivos endereços eletrônicos. Por essa razão, o referido banco de dados não pôde ser utilizado para distribuição dos questionários. A questão 1 foi definida como código do sujeito de pesquisa, solicitando-se nome e registro no Conselho Regional de Medicina (CRM) de cada participante, de modo que questionários cujos nomes não correspondessem ao CRM, bem como questionários em duplicidade e com nomes fantasiosos, foram excluídos. Sendo assim, a questão 1 não entra na apuração dos resultados, não sendo utilizados dados que possam identificar as informantes. A elaboração do questionário baseou-se em estudos semelhantes realizados por Umoetok et al. 5 e Kwong et al. 9 , adaptados para a realidade brasileira. A sequência das perguntas foi determinada de forma a proporcionar uma discussão coerente acerca dos resultados, sendo assim distribuídas: questão 1 coleta os dados cadastrais do sujeito de pesquisa; as questões 2 a 4 referem-se a dados demográficos; as questões 5 a 7 avaliam tempo de experiência e elementos da formação profissional; as questões 8 a 11 coletam dados sobre inserção no mercado de trabalho; as questões 12 a 14 avaliam o grau de envolvimento em posições de gestão e engajamento científico; e, por último, a questão 15 mensura a satisfação pessoal de cada participante enquanto mulher exercendo a especialidade. Após coleta, os dados foram tabulados em planilha do Microsoft Excel, para análise descritiva simples. O questionário aplicado, na íntegra, encontra-se descrito no Quadro 1 . Ressalta-se que este trabalho consiste em um estudo preliminar, desenvolvido no intuito de fomentar a discussão acerca de um tema atual e pouco discutido no meio vascular. Estima-se que atualmente a SBACV conte com cerca de 800 mulheres registradas no cadastro nacional da especialidade, de modo que o cálculo da amostra necessária para permitir inferência estatística está em torno de 260 questionários entre as associadas, considerando-se uma margem de erro máxima de 4% e nível de confiança de 95%. Uma pesquisa mais ampla está sendo elaborada, na qual será fundamental o apoio institucional da SBACV para que se atinja um número mais expressivo de participantes. 

**Quadro 1:** Questionário “Mulheres na cirurgia vascular: uma breve análise do perfil brasileiro”.

Questão 1. Informe seu nome e registro no CRM
Questão 2. Nacionalidade: ___Brasileira __outra (especifique____)
Questão 3. Estado onde atua: __Amazonas __Ceará __Pernambuco __ Alagoas __Paraíba __Sergipe __ Bahia __Tocantins __Goiás __Mato Grosso __Mato Grosso do Sul __Acre __Espírito Santo __Minas Gerais __Rio de Janeiro __São Paulo __Paraná __Santa Catarina __ Rio Grande do Sul __Distrito Federal __Amapá __Roraima __Rondônia __Pará __Rio Grande do Norte __Maranhão __Piauí
Questão 4. Qual a sua idade? __25-35 anos __36-45 anos __46-55 anos __56-65 anos __mais de 65 anos
Questão 5. Tempo de atuação na especialidade: __menos de 5 anos __6 a 10 anos __11 a 20 anos __mais de 20 anos
Questão 6. Cursou especialização? __Não, mas atuei como vascular e fiz prova de título depois __Sim, fiz residência médica __Sim, fiz estágio reconhecido pela SBACV __Ainda estou em treinamento __Nenhuma das anteriores
Questão 7. Possui algum destes títulos de especialista? __Título de especialista em angiologia __Título de especialista em cirurgia vascular __Área de atuação em ecografia vascular com Doppler __Área de atuação em angiorradiologia ou cirurgia endovascular __Possuo dois ou mais títulos acima __Não possuo título
Questão 8. Na sua prática atual, você atua... __Exclusivamente na rede pública __Exclusivamente na rede privada __Em ambas
Questão 9. Ocupa maior carga horária __Preferencialmente em consultório/clínica __Preferencialmente em hospital
Questão 10. Em que tipo de consultório/clínica você trabalha? __Próprio __Sublocado __Regime de parceria/percentual com proprietários de clínicas/consultórios __Outro (especifique)
Questão 11. Atualmente, qual o seu principal campo de atuação? __Cirurgia arterial __Cirurgia venosa __Cirurgia endovascular __Ecografia vascular __Flebologia estética __Atividades mescladas, não consigo especificar
Questão 12. Você já ocupou algum destes cargos de gestão? __Sim, dentro da SBACV __Sim, como preceptora de serviço __Não, nunca ocupei cargo de gestão __ Sim, outro cargo (especifique)
Questão 13. Já participou de algumas destas atividades científicas? __Apresentação em simpósio/congresso __Participação em mesa de simpósio/congresso __Ambos __Nenhum dos descritos acima
Questão 14. Já publicou trabalho científico? __Sim __Não
Questão 15. Em algum momento da carreira sentiu-se desvalorizada ou em desvantagem por ser mulher? ___Sim __Não

## RESULTADOS

 Decorridos 30 dias de coleta dos dados, dos 293 questionários enviados, 106 foram respondidos, dos quais cinco foram excluídos devido à inveracidade nos dados informados na questão 1. Dos 101 questionários válidos, apenas um relatou nacionalidade estrangeira (colombiana). A terceira pergunta (“Estado onde atua”) obteve maior número de respostas para São Paulo (n = 43), seguido do estado da Bahia (n = 11). A distribuição das participantes entre as cinco regiões brasileiras está ilustrada na Figura 1 . 

**Figura 1:** Gráfico ilustrando a distribuição das cirurgiãs vasculares por região no Brasil. Os valores estão em números absolutos.

 A questão 4 classificou as cirurgiãs vasculares quanto à faixa etária, estando 46,5% entre 25-35 anos de idade, 43,6% entre 36-45 anos, e menos de 10% acima dos 46 anos. Na pergunta 5, “Tempo de atuação na especialidade”, observou-se que 35,6% das participantes tinham menos de 5 anos de atuação na área, 36,6% tinham entre 6-10 anos, 23,8% entre 11-20 anos, e 4% tinham mais de 20 anos de profissão. 

 Na questão 6 (“Cursou especialização?”), 76,2% informaram ter feito residência médica, 18,8% fizeram estágio reconhecido pela SBACV, 4% ainda se encontravam em treinamento, e 1% respondeu “nenhuma das anteriores”. Os resultados para a pergunta de número 7 (“Possui algum destes títulos de especialista?”) estão ilustrados na Figura 2 . 

**Figura 2:** Gráfico ilustrando grau de especialização das participantes. Os valores estão em números absolutos. Note-se que a questão permite marcação em mais de uma resposta simultaneamente.

 Com relação aos aspectos econômicos do exercício profissional, obteve-se como resposta ao item 8 a atuação exclusiva na rede pública em 3 participantes, atuação exclusiva na rede privada em 29, e em ambas redes de saúde em 69. Na questão 9, 63,4% das participantes indicaram ocupar maior parte da carga horária preferencialmente em consultório/clínica. O item 10 avalia em que tipo de instituição é exercida a atividade ambulatorial, sendo que 39 participantes informaram ter negócio próprio, 24 trabalham em consultório sublocado, 41 sob regime de parceria/percentual com proprietários de clínicas/consultórios, e 11 responderam “outro”, especificando por escrita própria a atuação em ambulatório de convênio (n = 2), ambulatório do SUS (n = 2), residência médica (n = 2) e não tem consultório (n = 2). 

 Os resultados da pergunta 11 (“Atualmente, qual o seu principal campo de atuação?”) estão ilustrados na Figura 3 . 

**Figura 3:** Gráfico demonstrando a diversificação da participação feminina nos principais ramos de atuação dentro da especialidade. Os valores estão em números absolutos. Note-se que a questão permite marcação em mais de uma resposta simultaneamente.

 No item 12, “Já ocupou algum destes cargos de gestão?”, 7,9% responderam “sim, dentro da SBACV”; 30,7% já foram preceptoras de serviço; 5,9% já ocuparam outros cargos, discriminando por escrito a diretoria técnica de instituições de saúde (n=3); e 62,4% nunca ocuparam cargo de gestão. Os dados da questão 13 (“Já participou de alguma destas atividades científicas?”) estão dispostos na Figura 4 . No item 14, 64,4% das participantes mencionaram já haver publicado algum trabalho científico. 

**Figura 4:** Nível de engajamento científico das participantes. Os valores estão em números absolutos. Note-se que a questão permite marcação em mais de uma resposta simultaneamente.

 Por fim, na questão 15, “Em algum momento da carreira, sentiu-se desvalorizada ou em desvantagem por ser mulher?”, 64,4% das entrevistadas responderam que sim. 

## DISCUSSÃO

 Os dados levantados evidenciam que, na amostra avaliada, o perfil das cirurgiãs vasculares brasileiras é de mulheres de até 45 anos de idade que estão atuando na área por no máximo 10 anos. A baixa idade encontrada entre as entrevistadas corresponde ao interesse mais recente das mulheres pelas especialidades cirúrgicas, conforme comprovam os estudos de Troppmann et al. 11 . A história é marcada pela exclusão das mulheres, não somente no âmbito médico. Antropologicamente, os homens sempre foram considerados mais capazes para o uso de ferramentas e instrumentos de produção, o que lhes permitia a dominação sobre as mulheres 4 . Para atuar em uma carreira cirúrgica, impõe-se a necessidade de lidar com situações de muita gravidade, o que traduz uma tendência mundial a respeito da formação da nova geração de cirurgiões: jovens ávidos por situações desafiadoras e por um ambiente de trabalho onde possam expressar suas convicções 12 ^,^ 13 . Socialmente, existe uma predisposição e aceitação masculinas para desenvolver atividades de liderança e de combate, além de atividades que exijam maior esforço físico, o que pode ser encontrado na área cirúrgica 14 ^,^ 15 . Todas essas características foram culturalmente construídas ao longo de vários anos, resultando na ideologia de que os médicos do sexo masculino estariam mais aptos a ocuparem os cargos dentro da área cirúrgica 16 ; entretanto, esse perfil de profissional tem sido observado cada vez mais frequentemente entre as mulheres. 

 No presente estudo, houve a predominância de participantes do Sudeste, especialmente no estado de São Paulo, refletindo, em parte, a concentração dos centros formadores nessa região. A proporção de participantes é menor onde também há menor concentração de médicos especialistas. Sendo assim, as mulheres que se capacitam ajudam a compor a força de trabalho local, contribuindo para uma melhor distribuição de profissionais médicos pelo território nacional 17 . 

 Embora a maioria das cirurgiãs tenha cursado residência médica ou estágio reconhecido pela SBACV e mais da metade possua titulação junto às sociedades médicas profissionais, quase 2/3 nunca ocuparam cargo de gestão, sendo que aquelas que tiveram essa oportunidade estavam vinculadas a serviços de formação. Ao longo dos 66 anos de existência da SBACV, anteriormente denominada Sociedade Brasileira de Angiologia (SBA), apenas duas mulheres a presidiram: Dra. Merisa Garrido, entre os anos de 1990 e 1991, e Dra. Maria Elisabeth Rennó de Castro Santos, entre 2000 e 2001 18 . Além disso, nos últimos seis biênios, entre os 84 cargos, incluindo diretorias e vice-diretorias, apenas seis tiveram representatividade feminina, sendo, que nos últimos três mandatos nenhuma mulher esteve envolvida na gestão. A presença reduzida dessas mulheres nas diretorias da sociedade da especialidade corrobora com a percepção de haver uma sub-representatividade feminina nos cargos de liderança, evidenciando que, a despeito do aumento no número de cirurgiãs, seu avanço nas posições mais bem pagas e de alto prestígio permanece limitado 5 ^,^ 19 . Desvantagens como essas, que se impõem ao longo da trajetória profissional, foram percebidas por cerca de 64% das entrevistadas. 

 Em contrapartida, quase 2/3 das profissionais que responderam ao questionário informaram já ter publicado algum trabalho científico, e mais da metade indicou participação em congressos como palestrante ou integrante da mesa de debatedores. De fato, existe um engajamento nas atividades acadêmicas, que pode ser melhor desenvolvido se houver oportunidade de participação em pesquisas, publicações e apresentações ainda durante o período de treinamento em cirurgia vascular 20 . 

 A maior parte das cirurgiãs mescla atividades no serviço público e no privado, ocupando grande parte da sua carga horária em atendimentos ambulatoriais. Pouco mais de um terço das cirurgiãs vasculares possui negócio próprio, tendo a cirurgia venosa, a flebologia estética e a ecografia vascular como principal atividade. Apenas 12 participantes indicaram ter a cirurgia arterial como principal campo de atuação e somente 10 se dedicavam prioritariamente à cirurgia endovascular. Sabe-se que, dentre os fatores relacionados ao pequeno número de mulheres na cirurgia arterial e endovascular, estão as dificuldades em conciliar a profissão com a vida pessoal, incluindo casamento e maternidade 21 . Soma-se a isso o entendimento de que a definição do campo de atuação dentro da especialidade nem sempre é uma questão de escolha. Não se pode deixar de mencionar que a preferência pela incorporação de profissionais do sexo masculino aos grupos de cirurgia arterial constitui uma barreira cultural a ser vencida. Como relatado por Franco e Santos 4 , características pessoais tidas como necessárias para a atuação dentro da área cirúrgica, como liderança, autocontrole, capacidade de questionamento, personalidade forte e algum grau de agressividade, são vistos como inerentes ao sexo masculino e estranhos à personalidade feminina. 

 Por outro lado, levando em conta a incorporação de novas tecnologias no campo da flebologia, a escolha pela cirurgia venosa e estética, além de uma rota de fuga, já que esse ramo possibilita conciliar qualidade de vida e flexibilidade de horários, pode configurar uma estratégia promissora de integração feminina ao mercado de trabalho. A expansão do campo da flebologia, uma vez que requer atualização e capacitação contínuas, impulsiona a emergência de novos postos de liderança científica, que podem perfeitamente ser ocupados por mulheres. Assim, com boas perspectivas de crescimento acadêmico e financeiro, há o potencial incentivo para a promoção de carreiras de sucesso, revertendo-se o paradigma apontado pela última questão do presente estudo. 

 Mundialmente, o entendimento acerca da existência de fatores que limitam a plena inserção da mulher nas carreiras cirúrgicas vem gerando alguns movimentos no sentido de incentivar a integração e cooperação mútua entre cirurgiãs, como a criação da Women in Thoracic Surgery (WTS) 22 e da Association of Women Surgeons (AWS) 23 . No Brasil, a Sociedade Brasileira de Cirurgia Cardiovascular (SBCCV) tem desenvolvido projetos motivacionais como o vídeo *Ballet de Mãos – As Cirurgiãs Cardiovasculares* 24 , que valoriza e enaltece a presença feminina dentro da especialidade. Embora ainda sejam incipientes, propostas como essas podem se tornar a força motriz de mudanças no cenário da mulher nas especialidades cirúrgicas. 

## LIMITAÇÕES

 O número de participantes entrevistadas no presente estudo é pequeno (n = 101), frente à população de cirurgiãs vasculares brasileiras. Dessa forma, os resultados aqui apurados não podem ser generalizados, aplicando-se apenas à amostragem selecionada. Além disso, houve um viés de seleção, uma vez que envio dos questionários não abrangeu a totalidade das cirurgiãs vasculares do país, pois participaram da pesquisa apenas as mulheres que faziam parte da rede de contatos das autoras. 

## CONCLUSÃO

 O futuro da cirurgia vascular depende da formação de profissionais qualificados, de ambos os gêneros. Entretanto, considerar o impacto da participação feminina nessa força de trabalho é de fundamental importância na elaboração de estratégias para o bom desempenho da especialidade nas gerações futuras. O presente estudo corrobora com a ideia de que as cirurgiãs vasculares demonstram uma contínua dedicação ao exercício da especialidade, bem como almejam crescimento acadêmico. Embora o trabalho apresentado seja um estudo preliminar, acredita-se que ele abra um precedente para que novas pesquisas acessem detalhadamente os pormenores da prática profissional das cirurgiãs vasculares, numa amostragem mais ampla e elegível à inferência estatística, fomentando a discussão acerca das características e das desigualdades entre os gêneros dentro da especialidade, como tem sido tendência mundial em diversos outros campos do conhecimento médico.
